# Supplementary material for: Complexome profiling of the Chlamydomonas psb28 mutant reveals TEF5 as an early PSII assembly factor
Source: Plant Cell. 2025 Mar 17;37(6):koaf055. doi: 10.1093/plcell/koaf055 (PMC12164590; doi:10.1093/plcell/koaf055)
Supplement: koaf055_Supplementary_Data [file koaf055_supplementary_data.zip › psb28_interactive.zip!/plots/intensity/qualityPlots/peptides/MAS1_Cre03.g144807.t1.1.html]

### Cre03.g144807.t1.1

Trivial name: MAS1  
SeqCoverage [median]: 23.80  
Mass: 60.71 kDa  
  
 

| Sequence | MissedCleavage | Mass [Da] | wtIntensity | muIntensity | Score |
| --- | --- | --- | --- | --- | --- |
| DLDAGRLPDFLPETAAVR | 1 | 1955.01 | 431.78 | 0.00 | 32.09 |
| GGALLAEGGITEK | 0 | 1214.65 | 12070.83 | 23483.49 | 113.22 |
| GVHAMGGMAAQIPIKDDPAANAAALAK | 1 | 2588.32 | 0.00 | 7869.25 | 113.42 |
| HMPTPNQLHVR | 0 | 1328.68 | 567.89 | 794.49 | 54.49 |
| NHPQFVLPDR | 0 | 1221.63 | 7513.97 | 9889.69 | 97.46 |
| QKDLDAGRLPDFLPETAAVR | 2 | 2211.16 | 0.00 | 3236.07 | 38.89 |
| RDDVTVTAHNLLDVR | 1 | 1722.90 | 5240.84 | 12315.43 | 73.89 |
| RVEITGPVDR | 1 | 1140.63 | 0.00 | 658.30 | 52.69 |
| RVEITGPVDRK | 2 | 1268.72 | 316.81 | 0.00 | 59.57 |
| SAVTMTSPFMDAYVR | 0 | 1674.77 | 3508.66 | 5809.93 | 99.50 |
| SAVWQWVR | 0 | 1030.53 | 0.00 | 679.75 | 79.12 |
| VEITGPVDR | 0 | 984.52 | 540.93 | 815.57 | 78.15 |
| VEITGPVDRK | 1 | 1112.62 | 8935.87 | 9789.57 | 87.79 |
|  |  |  | 39127.6 | 75341.5 |  |
